# Genotyping of a gene cluster for production of colibactin and in vitro genotoxicity analysis of *Escherichia coli* strains obtained from the Japan Collection of Microorganisms

**DOI:** 10.1186/s41021-020-00149-z

**Published:** 2020-03-11

**Authors:** Masanobu Kawanishi, Chiaki Shimohara, Yoshimitsu Oda, Yuuta Hisatomi, Yuta Tsunematsu, Michio Sato, Yuichiro Hirayama, Noriyuki Miyoshi, Yuji Iwashita, Yuko Yoshikawa, Haruhiko Sugimura, Michihiro Mutoh, Hideki Ishikawa, Keiji Wakabayashi, Takashi Yagi, Kenji Watanabe

**Affiliations:** 1 Graduate School of Science and Radiation Research Center, Osaka Prefecture University, 1-2 Gakuen-cho, Naka-ku, Sakai-shi, Osaka, 599-8570 Japan; 2 Department of Pharmaceutical Sciences, University of Shizuoka, Shizuoka, Japan; 3 Graduate Division of Nutritional and Environmental Sciences, University of Shizuoka, Shizuoka, Japan; 4 Department of Tumor Pathology, Hamamatsu University School of Medicine, Shizuoka, Japan; 5 School of Veterinary Medicine, Faculty of Veterinary Science, Nippon Veterinary and Life Science University, Tokyo, Japan; 6 Division of Prevention, Center for Public Health Sciences, National Cancer Center, Tokyo, Japan; 7 Department of Molecular-Targeting Cancer Prevention, Kyoto Prefectural University of Medicine, Kyoto, Japan

**Keywords:** Colibactin, Genotyping, Genotoxicity

## Abstract

**Introduction:**

Colibactin is a small genotoxic molecule produced by enteric bacteria, including certain *Escherichia coli* (*E. coli*) strains harbored in the human large intestine. This polyketide-peptide genotoxin is considered to contribute to the development of colorectal cancer. The colibactin-producing (*clb*^+^) microorganisms possess a 54-kilobase genomic island (*clb* gene cluster). In the present study, to assess the distribution of the *clb* gene cluster, genotyping analysis was carried out among *E. coli* strains randomly chosen from the Japan Collection of Microorganisms, RIKEN BRC, Japan.

**Findings:**

The analysis revealed that two of six strains possessed a *clb* gene cluster. These *clb*^+^ strains JCM5263 and JCM5491 induced genotoxicity in in vitro micronucleus (MN) tests using rodent CHO AA8 cells. Since the induction level of MN by JCM5263 was high, a bacterial *umu* test was carried out with a cell extract of the strain, revealing that the extract had SOS-inducing potency in the *umu* tester bacterium.

**Conclusion:**

These results support the observations that the *clb* gene cluster is widely distributed in nature and *clb*^+^*E. coli* having genotoxic potencies is not rare among microorganisms.

## Introduction

Colibactin is a small genotoxic molecule produced by *Enterobacteriaceae*, including certain *Escherichia coli* (*E. coli*) strains harbored in the human gut, and is involved in the etiology of colorectal cancer. The colibactin-producing (*clb*^+^) microorganisms possess a 54-kilobase genomic island (*clb* gene cluster) encoding polyketide synthases (PKSs), nonribosomal peptide synthetases (NRPSs), and PKS-NRPS hybrid megasynthetases [1]. Nougayrede et al. observed DNA double-strand breaks and interstrand cross-links in human cell lines and in animals infected with *clb*^+^*E. coli* strains*,* resulting in generation of gene mutations [1]. The *clb*^+^*E. coli* stimulates growth of colon tumors under conditions of host inflammation, and is found with increased frequency in inflammatory bowel disease, familial adenomatous polyposis, and colorectal cancer patients [2, 3]. We previously reported that *E. coli* strains isolated from a Japanese colorectal cancer patient produced colibactin and showed genotoxicity in in vitro assays [4, 5]. However, the chemical structure of the genotoxin, the molecular mechanism of its mutagenesis/carcinogenesis, and distribution of the *clb* gene cluster among microorganisms have not been fully clarified yet.

The present study aimed to assess the distribution of the *clb* gene cluster among *E. coli* strains randomly chosen from the Japan Collection of Microorganisms, with genotyping of the gene cluster. To evaluate the association between presence of the cluster and genotoxicity, we examined the genotoxicity/clastogenicity of these *E. coli* strains in rodent cells using the in vitro micronucleus (MN) test. Using the *umu* test, DNA damage in a bacterial tester strain treated with crude extracts of the *E. coli* was also evaluated.

## Materials and methods

### *E. coli* strains and genotyping

Six *E. coli* strains (*Escherichia coli* (Migula 1895) Castellani and Chalmers 1919) were randomly chosen and purchased from the Japan Collection of Microorganisms at the microbe division of the RIKEN BioResource Research Center (Tsukuba, Japan), which is participating in the National BioResource Project of the MEXT, Japan. *E. coli* Nissle 1917 strain was obtained from Mutaflor, Ardeypharm, GmbH. (Herdecke, Germany), and used as a *clb*^+^ strain [1]. The host tester strain *E. coli* ZA227 used in the *umu* test was kindly supplied by Dr. Mie Watanabe-Akanuma (Institute of Environmental Toxicology, Tokyo, Japan). PCR analysis and electrophoresis for genotyping of the *clb*^+^ gene cluster was carried out with the oligonucleotide primers, as previously reported [4]. For genome analysis with next-generation sequencing, the *E. coli* genomic DNA was purified with MonoFas DNA Purification Kit V (GL Sciences In., Tokyo, Japan). Library construction and paired-end sequencing were carried out using the Miseq (Illumina Inc., San Diego, CA, U.S.A) with the Miseq reagent kits v2 (300 cycles). The raw sequence data were mapped by the HISAT2 program (ver. 2.1.0, Johns Hopkins University, Baltimore, MD, U.S.A) to the genome of Nissle1917 (GCA_000714595.1) as a reference sequence. The mapped files were converted to bam files by using SAMtools (ver1.9, http://www.htslib.org), and the read coverages were generated by StringTie (ver1.3.5, Johns Hopkins University) and the heatmap was constructed using the CIMminer program (National Cancer Institute, Bethesda, MD. U.S.A).

### Infection and in vitro micronucleus test

Bacterial infection to Chinese hamster ovary (CHO) AA8 cells and the MN test were carried out as previously described [5]. Briefly, the CHO cells (4 × 10^5^ cells/dish) were seeded in ϕ60 mm plastic cell culture dishes 1 day before the infection procedure. The bacteria were cultured until OD_595_ = 0.5 at 37 °C in Infection Medium (IM) (RPMI1640 medium (Nacalai Tesque., Kyoto, Japan) + 25 mM HEPES, 5% fetal bovine serum (FBS, Sigma-Aldrich, MO USA)). The infection was carried out with 3 mL of IM containing *E. coli* at the indicated multiplicity of infection (MOI) (number of bacteria per cell at the onset of infection). After being treated with bacteria for 4 h, the CHO cells were cultured for a further 20 h in cell culture medium supplemented with 200 μg/mL gentamicin (Nacalai Tesque). The MN test was then performed, and the number of cells with MN was recorded based on the observation of 1000 interphase cells. Relative cell growth was calculated using the formula:

$$ \mathrm{Relative}\ \mathrm{cell}\ \mathrm{growth}=\left(\mathrm{number}\ \mathrm{of}\ \mathrm{treated}\ \mathrm{cell}\mathrm{s}\right)\div \left(\mathrm{number}\ \mathrm{of}\ \mathrm{non}-\mathrm{treated}\ \mathrm{cell}\mathrm{s}\right) $$

### *umu* test

The DNA damaging potency of bacterial cell extracts was estimated using the *umu* test, as previously described [5]. Briefly, *E. coli* cells were harvested from 10 mL of overnight culture in LB media (O.D. = 1.7–2) by centrifugation, and extracts of *E. coli* were prepared with 1 mL of BugBuster protein extraction reagent (Novagen, Merck Millipore Co., Tokyo, Japan). The cell lysates were collected by centrifugation at 16,000×g for 20 min at 4 °C. The *umu* assay using ZA227/pSK1002 tester strain was conducted as previously reported [6, 7]. ZA227 is derived from *E. coli* K-12, which dose not posses the *clb* gene cluster [1]. The tester strain in 1 mL of the TGA medium and 20 μL of the extracts from the *clb*^+^*E. coli* strains were incubated for 3 h at 37 °C. As a solvent and positive controls, 20 μL of BugBuster solution and 10 μL of 1 μg/mL 4-nitroquinoline 1-oxide (4-NQO) (Nacalai Tesque) were used, respectively.

## Results and discussion

### Genotyping

**Fig. 1:**
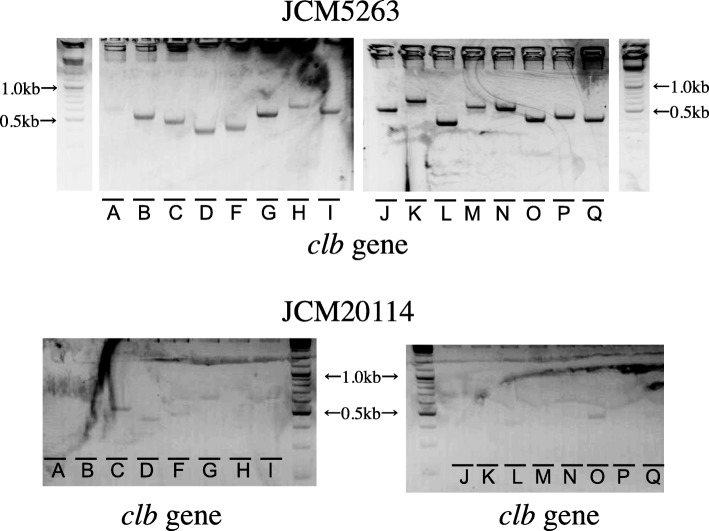
Typical gel images of amplicons from genomic DNA of a *clb*^+^ and a *clb*^−^ strain. Genomic DNA of JCM5263 (*clb*^*+*^) and JCM20114 (*clb*^*−*^) were analyzed. The *clb* genes and expected sizes of their amplicons (bp) in PCR are as follows: *clbA*, 613; *clbB,* 555; *clbC*, 503, *clbD*, 431; *clbF*, 465; *clbG*, 599; *clbH*, 693; *clbI*, 643; *clbJ*, 544; *clbK*, 690; *clbL*, 401; *clbM*, 592; *clbN*, 581; *clbO*, 438; *clbP*, 464; *clbQ,* 430

**Fig. 2:**
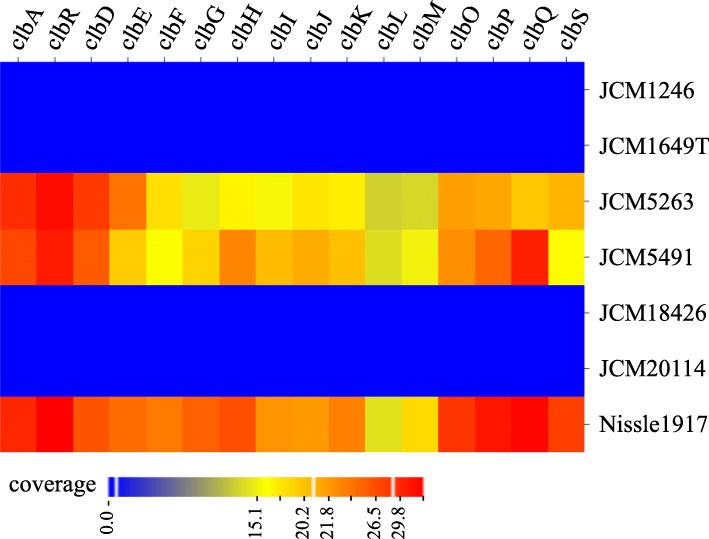
The read coverage of the *clb* genes in genomic DNA of *E. coli* strains determined by Illumina MiSeq. The color represents the read coverage of the indicated *clb* gene in the indicated strains

First, we assessed the presence of the *clb* genes, i.e., 16 *clb* genes (*clbA-clbD, clbF-clbQ*) by detecting each amplicon after PCR with specific primer sets to the genes. As a positive control strain, we analyzed the known *clb*^+^ strain Nissle 1917, which is a commensal strain also widely used as a probiotic treatment for intestinal disorders [1]. In genomic DNA from JCM5263, JCM5491 and Nissle 1917, we observed amplicons corresponding to all 16 *clb* genes (Table 1 and Fig. 1). However, some or all of the amplicons corresponding to the 16 genes were not detected in JCM1649T, JCM1246, JCM18426 and JCM20114. The presence or absence of the *clb* gene cluster in the strains was also confirmed with next-generation sequencing of the bacterial genomic DNA (Fig. 2). We concluded that three among seven strains, i.e., six strains randomly chosen from the Japan Collection of Microorganisms and the positive control strain Nissle 1917, harbored the *clb* gene cluster (Table 1). It has been reported that 20.8% of healthy people who have neither inflammatory bowel disease nor colorectal cancer as well as 66.7% of colorectal cancer patients harbor *clb*^+^*E. coli* [8]. Furthermore, this gene cluster is found not only in *E. coli* but also in *Klebsiella pneumoniae*, *Enterobacter aerogenes* and *Citrobacter koseri* [8, 9]. The bacterial *clb* gene cluster seems to be well-distributed in nature.

**Table 1:** Summary of genotyping and genotoxicity analyses

Strain	*clb* Gene cluster	Genotoxicity (MN test)
JCM1246	–	–
JCM1649T	–	–
JCM5263	+	+
JCM5491	+^a^	+
JCM18426	–	–
JCM20114	–	–
Nissle 1917	+^b^	+

^a^Genotyping data are also confirmed in ref. [4]

^b^Genomic data are also from ref. [1]

### In vitro genotoxicity analysis

**Fig. 3:**
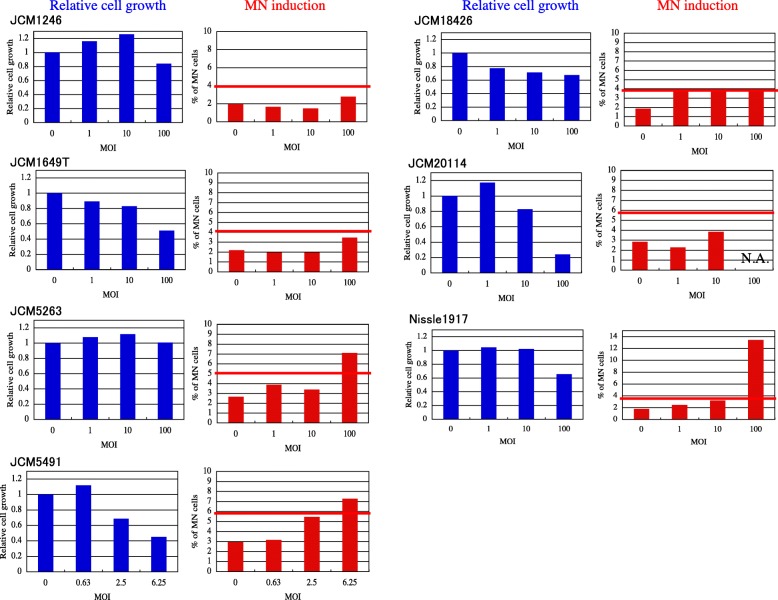
Micronuclei formation in CHO AA8 cells infected with *clb*^*+*^*E. coli*. Relative cell growth and mean values of MN frequencies at least 1000 cells are shown. In the graph, MOI = 0 represents the vehicle control (treatment with IM). Horizontal red lines in MN graphs indicate MN frequencies two fold higher than those of each vehicle control. N.A. indicates data are not available due to the high cytotoxicity

**Fig. 4:**
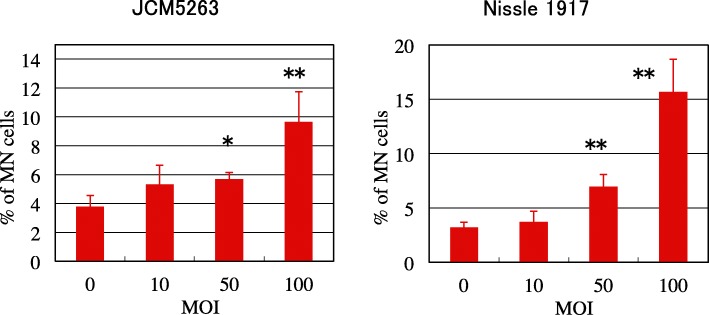
Dose-dependent induction of micronuclei in CHO AA8 cells infected with *clb*^*+*^*E. coli*. Mean ± SD values of at least three independent experiments are shown. MOI = 0 represents the vehicle control. * indicates *p* < 0.05 and ** indicates *p* < 0.01 (versus that of MOI = 0) according to the *t*-test

Next, for screening of their genotoxicities, the MN-inducing activity of the *E. coli* strains was examined using the CHO AA8 cell line, since the test is a convenient and reliable for evaluating genotoxicity [10]. As shown in Fig. 3, the degree of induction varied among the strains. In the present study, we determined that *E. coli* induces MN-frequency at least twofold compared with MOI = 0 as an MN-induction positive strain. Evidently, JCM5263 and Nissle 1917 were MN-induction positive strains, that is, infection of both strains at MOI = 100 induced MN with frequency 2.5- to 7-fold greater than that at MOI = 0. The level of cytotoxicity also varied. Infection of JCM5491 and JCM20114 led to high cytotoxic effects in CHO cells. The relative growths of CHO cells treated with JCM5491 and JCM20114 at MOI = 100 were 2.6% (data not shown) and 24%, respectively. JCM5491 and JCM20114 were hemolysin-positive strains (data not shown), therefore, their high cytotoxicity might be involved in hemolysin. Since the MN test cannot be performed under such highly-cytotoxic conditions, we tried lower-MOI treatments and found that at MOI = 6.25, JCM5491 induced MN with frequency 2.5-fold greater than that at MOI = 0 (Fig. 3). We concluded that *clb*^+^ JCM5263, JCM5491 and Nissle 1917 are MN-induction positive strains (Table 1). We also confirmed that infections with JCM5263 and Nissle 1917 resulted in dose-dependent MN-inductions (Fig. 4).

**Fig. 5:**
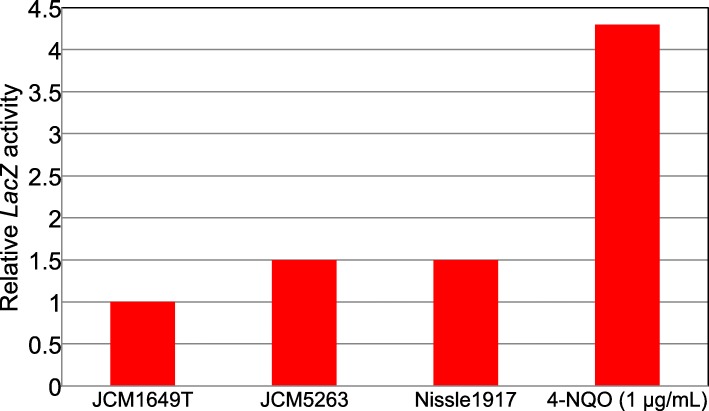
Induction of SOS response (*umuC* gene) by *E. coli* extracts in *umu* test. Relative *LacZ* activity to the *clb*^−^ strain JCM1649T. Mean values of duplicated determinations are shown. 4-NQO as a positive control of DNA damaging agent (incubated for 3 h at 37 °C)

Since DNA damage is known to induce MN [10], we examined the extracts of *clb*^*+*^*E. coli* (JCM5263 and Nissle 1917) for induction of an SOS response in the *umu* test. The extracts were prepared using BugBuster reagent, which disrupts the cell walls and liberates the cytosol. Increased SOS responses were observed in the extracts of *clb*^*+*^ from both JCM5263 and Nissle 1917 compared with that of *clb*^*−*^ JCM1649T (Fig. 5). The relative SOS-induction levels by the extracts of both JCM5263 and Nissle 1917 were 1.5 times higher than that of JCM1649T. The induction level by the positive control agent 4-NQO (1.0 μg/mL) was 4.3-fold that by JCM1649T. These results indicate that the *clb*^*+*^*E. coli* extracts have weak potency for SOS induction. The *clb*S gene encodes a resistance protein blocking the genotoxicity of colibactin and ClbS protein functions as an antidote for colibactin-autotoxicity in *clb*^*+*^*E. coli* [11]. Presumably, the presence of ClbS protein in the extracts in the present study attenuated their DNA-damaging potency.

## Conclusion

Genotyping analysis revealed that two of six *E. coli* strains randomly chosen from the Japan Collection of Microorganisms possessed a *clb* gene cluster. The *clb*^+^ JCM5263, JCM5491 and Nissle 1917 (as *clb*^+^ control strain) exhibited MN induction in CHO cells. The cell extracts of JCM5263 and Nissle 1917 also had DNA-damaging potency in a bacterial *umu* test. These results support the observations that *clb* gene clusters are widely distributed in nature and that *clb*^+^*E. coli,* which has genotoxic potency, is not rare among microorganisms.

## Data Availability

Not applicable.

## Abbreviations

4-NQO: 4-nitroquinoline 1-oxide
CHO: Chinese hamster ovary
clb^+^: colibactin-producing
MN: micronucleus
MOI: multiplicity of infection
